# The Relation Existing between General Digestive Conditions and Mouth Conditions

**Published:** 1920-05

**Authors:** M. Marion Null


### THE RELATION EXISTING BETWEEN GENERAL DIGESTIVE CONDITIONS AND MOUTH CONDITIONS

BY M. MARION NULL, M.D.

The teeth and gums are merely a part of the digestive machine and help to do a portion of the mechanical work of preparing the food for assimilation. That the digestion of the average person is dependent in large measure upon the condition of the teeth has long been recognized by both physician and dentist, and also the relationship of the whole digestive system upon the mouth; but neither the physician nor the dentist, in my opinion, up to the present time, has given these conditions enough consideration in the treatment of their patients.

That there exists a relationship between the diseases of the teeth and mouth and the diseases of the stomach and intestines none can dispute. In the medical and dental schools we have been taught that bad teeth, poor mastication and infected gums are a cause of digestive disturbances. We have thought only of one side, one part of the relationship of the physiology of digestion.

I am convinced that diseases of the stomach and intestines, constitutional diseases, and a faulty, unbalanced food, may profoundly effect the teeth and mouth and produce caries of the teeth and pyorrhea alveolaris. Many patients consult a physician when they need a dentist, and a dentist when they need a physician. It is the sifting process, the getting down to the real cause of the troubles and thereby benefiting the patient the most, that constitutes the central thought of this paper.

A patient came to me a few days ago for stomach trouble. He had typical symptoms. He had been seen by several physicians and in spite of treatment had steadily grown worse. A dentist had only a few weeks before finished working on his teeth. As his stomach symptoms had been present only a short time and as he had the appearance of having been sick a long time, I looked for the cause elsewhere. The leucocyte count was high. The X-ray revealed the cause, and he was sent to a dental surgeon to have every tooth extracted, as he had pus in both antrums and pus pockets about his teeth. In a few days he was at work again. The mouth in this case was clearly the cause of the trouble with his stomach.

Again, a young lady came to me with a very sore tongue and mouth which had resisted treatment for four years. She complained of nothing else except her teeth. They had been filled and refilled while several were lost and replaced by bridgework. A careful examination revealed an atonic condition of the stomach with a deficient amount of gastric secretion; even litmus showed acid in the mouth. After the correction of the stomach trouble the mouth healed without any local treatment and has remained so for months. Had the stomach condition been recognized sooner she would have had a good set of teeth today. In this case the stomach was clearly the cause of the trouble with the mouth and teeth.

To make my position perfectly clear, let me say that acid conditions of the mouth and all mouth pathology due to acid foods or medicines, fungi and decomposing food material are purposely omitted in this discussion. Every physician and dentist recognizes these conditions. I have in mind only those cases where the condition of the mouth causes dyspeptic symptoms, or where stomach diseases produce pathology in the mouth.

I have been interested recently in studying the mouths and tongues of patients. From time immemorial the tongue has been considered the ''index'' of the digestive system. It was the first thing the old doctor of our fathers used to examine. And I must say that in the light of what I have found that I do not consider the looking at the tongue old-fashioned for either physician or dentist. A ''coated tongue'' may come from two causes—local or general. The local causes are infections of the mouth, nose, throat or tonsils, which can be plainly seen and which does not concern us here. It is infrequent in comparison with the general cause.

Now the general cause of a ''coated tongue'' is the condition of the stomach and intestinal tract. The stomach is a mere dilation of the digestive tube. If you remember the digestive canal in early fetal life is a straight tube of even diameter, and the size and length depend on the uneven growth in various areas as the child develops. The upper outlet of the stomach, the cardia, has a weak sphincter muscle shutting it off from the oesophagus.

A ''coated tongue'' is therefore an extension of the condition in the stomach up the oesophagus and on to the base of the tongue. There is a retrograde movement of particles of food from the stomach up the oesophagus. To prove this condition I took lycopodium powder and put it in gelatine capsules. These were brushed and washed off till not a particle of powder remained on the outside. If these powders were swallowed at night lycopodia powder could be taken off the tongue of most people in the morning, and so recognized under the microscope.

In certain diseased conditions it was worse than others, but in most individuals more or less could be found. In some cases the powder was found on the tongue in sixty to ninety minutes. Other insoluble powders have been used with the same results. Dr. Kast, of New York City, has carried out a similar line of experiments with similar results. This experiment shows conclusively that there is an upward movement of particles of food from the stomach to the mouth, that the rough part of the tongue catches this food with mucus and bacteria, and this forms a breeding place for germs with formation of acids of decomposition. As this lies in the close proximity to the teeth we have the pathological setting for teeth involvement.

This retrograde movement is found in some people with seemingly no diseased condition of the stomach, but rather in the infantile type of gastric anatomy; for in the infant the stomach lies almost perpendicular and assumes the transverse position as the child grows older. A "coated tongue" is generally found in a low acid condition of the stomach, as acid in the stomach closes the cardiac sphincter tightly; atonic stomach; retention in the stomach; chronic catarrh of the intestines; cancer; appendicitis and gallstones.

Seldom a day will pass that the dentist with an ordinary practice will not have in his chair some of the preceding types for patients. If he is alert and if he wants to do the best by his patient, in fact if he wants to fortify his own work, it stands him in hand to notice the condition of the patient's tongue, mouth and general health, and if questionable to send him to a physician.

Patients have come to me vexed with their dentist because their work has not lasted, when in fact the dental work was an excellent job. The only criticism that could be offered against the dentist was that he failed to recognize that the patient needed a physician and to have sent him to have his digestive disturbances corrected.

Almost all of the patients with digestive disturbances who come to my office I am having X-ray plates taken of their teeth. Twenty-two per cent. have serious pathological conditions in their mouths. The symptoms of one woman who had worn full plates for years, not knowing that she had a tooth in her mouth, all disappeared when an unerupted third molar had been extracted.

Some years ago Dr. J. H. Kellogg made a study of 100 cases, reporting on the condition of the teeth with special reference to the digestive organs. Of the 100 cases studied only two had perfect teeth. Of those suffering from the deficiency of gastric acid, about 50 out of the 100, 10 per cent. had fair teeth, while of those suffering with hyperacidity, 20 per cent. had good teeth. Of those suffering from a simple dyspepsia none had artificial teeth, while of the 51 suffering from deficiency of gastric acid 12 had artificial teeth, five had upper set only and seven both upper and lower sets. Of those suffering from gastric catarrh the number of sound teeth was only one-half as many as in persons not so affected. These facts seem to show that there is an undisputed relationship between diseases of the stomach and dental caries.

In conclusion, let me say that the dentist who notices the "coated tongue," the bad breath, the dingy complexion, constipation, and a mouth full of decaying teeth will benefit his patient materially, if he not only treats one part of the trouble—the teeth—but sends him to a physician who is competent to correct the digestive disturbance. He will thereby fortify his own work and gain the patient's everlasting gratitude.—*Northwest Journal of Dentistry.*

